# Liver Transplantation for Hepatocellular Carcinoma With Bile Duct Tumor-Associated Thrombi: A Systematic Review and Pooled Analysis

**DOI:** 10.3389/frtra.2022.879056

**Published:** 2022-04-29

**Authors:** Steven C. Kim, Alexandra C. Bolognese, Christopher J. Little, Mary E. Hitchcock, Glen E. Leverson, David P. Al-Adra

**Affiliations:** ^1^Division of Transplantation, Department of Surgery, University of Wisconsin School of Medicine and Public Health, Madison, WI, United States; ^2^Ebling Library, University of Wisconsin, Madison, WI, United States; ^3^Department of Surgery, University of Wisconsin School of Medicine and Public Health, Madison, WI, United States

**Keywords:** bile duct-associated tumor thrombi (BDTT), liver transplantation, hepatocellular carcinoma (HCC), survival, tumor recurrence

## Abstract

**Introduction:**

The significance of bile duct tumor-associated thrombi in patients undergoing transplantation for hepatocellular carcinoma (HCC) is controversial. Therefore, we performed a systematic review of the literature with pooled analysis to investigate the impact of biliary invasion on HCC recurrence and patient survival.

**Methods:**

Of 1,584 references screened, eight were included for analysis. Demographics, patient and tumor factors, recurrence, and survival data were analyzed. Time to recurrence and death were extracted from each paper by cross-referencing survival curves.

**Results:**

A total of 35 patients across eight studies were pooled for analysis when follow-up data were available. At 1 year, 92.9% of patients undergoing transplantation for HCC with bile duct thrombi were alive. Overall survival at 3 and 5 years was 65.5 and 49.6%, respectively. At 1 year, 21.6% of patients had recurrence of their disease, while at 3 years, 50.4% of patients had recurrence. Of those patients with recurrence in the first year, 71.4% recurred within the first 3 months after transplantation.

**Conclusion:**

Overall patient survival decreased within the first 5 years, but then stabilized. The 5-year survival rate of 49.6% in this pooled analysis is lower than that reported for patients undergoing transplantation for HCC within the Milan criteria (50–78%) or recent reports in patients with portal vein involvement (63.6%), though data is limited by a lack of long-term follow-up in this understudied population. Transplantation for patients with HCC with bile duct involvement may be a viable treatment option, warranting further investigation.

## Introduction

In cirrhotic patients with hepatocellular carcinoma (HCC), liver transplantation provides an opportunity for curative treatment of both disease processes. Inclusion criteria vary between centers when considering liver transplantation for patients with HCC. While the Milan criteria are most commonly used, other criteria such as the UCSF criteria and the extended Toronto criteria have led to more aggressive parameters for transplantation (1–3). Examination of liver explant specimens in the setting of these expanded criteria has facilitated the association of some pathologic characteristics with poorer recurrence and survival outcomes, including the presence of macro- and microvascular invasion (4, 5).

Bile duct tumor thrombus in the setting of HCC is a relatively unstudied phenomenon, likely because of its low incidence (reported as 0.5–12.9% of surgical specimens). Most of the limited data available come from a systematic review of patients undergoing liver resection and suggest that resection can be safely performed in patients with HCC and BDTT without a decrease in overall survival and or an increase in postoperative recurrence (6). Although the prognostic factors for both overall survival and risk of recurrence for HCC treated with liver transplantation have been well-described, few studies have investigated the impact of bile duct-associated tumor thrombi (BDTT) on outcomes. Further, these studies have primarily been limited to single-institution case-series and case reports. One prior meta-analysis examined prognosis in patients with HCC with BDTT, but only one of the 11 reviewed studies included patients undergoing liver transplantation as therapy (7). This scarcity of data has led to disagreement in the transplant community regarding the candidacy for liver transplantation for HCC with BDTT, and no consensus currently exists regarding the preferred treatment modality in this setting (8).

We performed a systematic review of the published literature with pooled analysis to examine the impact of BDTT on tumor recurrence and overall survival in patients undergoing liver transplantation for HCC.

## Materials and Methods

### Inclusion Criteria for Considering Studies for This Review

#### Study Characteristics

Given the rarity of HCC with BDTT being treated with transplantation, studies with one or greater patients were included to ensure comprehensive capture of the available clinical experience. This included human case reports (1 case), case-series (>1 case), randomized controlled trials, non-randomized controlled trials, and prospective cohort series.

#### Participants

The target population consisted of adult (>18 years old) male or female patients undergoing liver transplantation for HCC who were found to have BDTT on final pathological specimen.

#### Search Terms

Using a keyword search for relevant terms in PubMed, Scopus, and Cochrane, references were imported into Covidence (https://www.covidence.org/) for screening (Supplementary Figure 1). Studies were screened by three reviewers independently.

### Data Extraction and Management

Survival and recurrence data were extracted and analyzed from the included studies. Months to recurrence and months to death or last known follow-up per patient were abstracted from each paper either directly from figures or text, or by cross-referencing survival and recurrence curves from each reference.

### Statistical Analysis

Pooled analysis was performed on the data from included studies. Descriptive statistics (simple counts, means, and medians) were used to report patient and tumor related data. Due to the lack of randomized controlled trials, a meta-analysis was not deemed appropriate. Overall survival and recurrence analysis was performed using SAS Software (SAS Institute, Cary, NC). Kaplan-Meier curves were generated for both overall survival and recurrence-free survival. If a patient did not have recurrence or time to death data available, they were excluded from the analysis.

## Results

### Results of the Search

**Figure 1:**
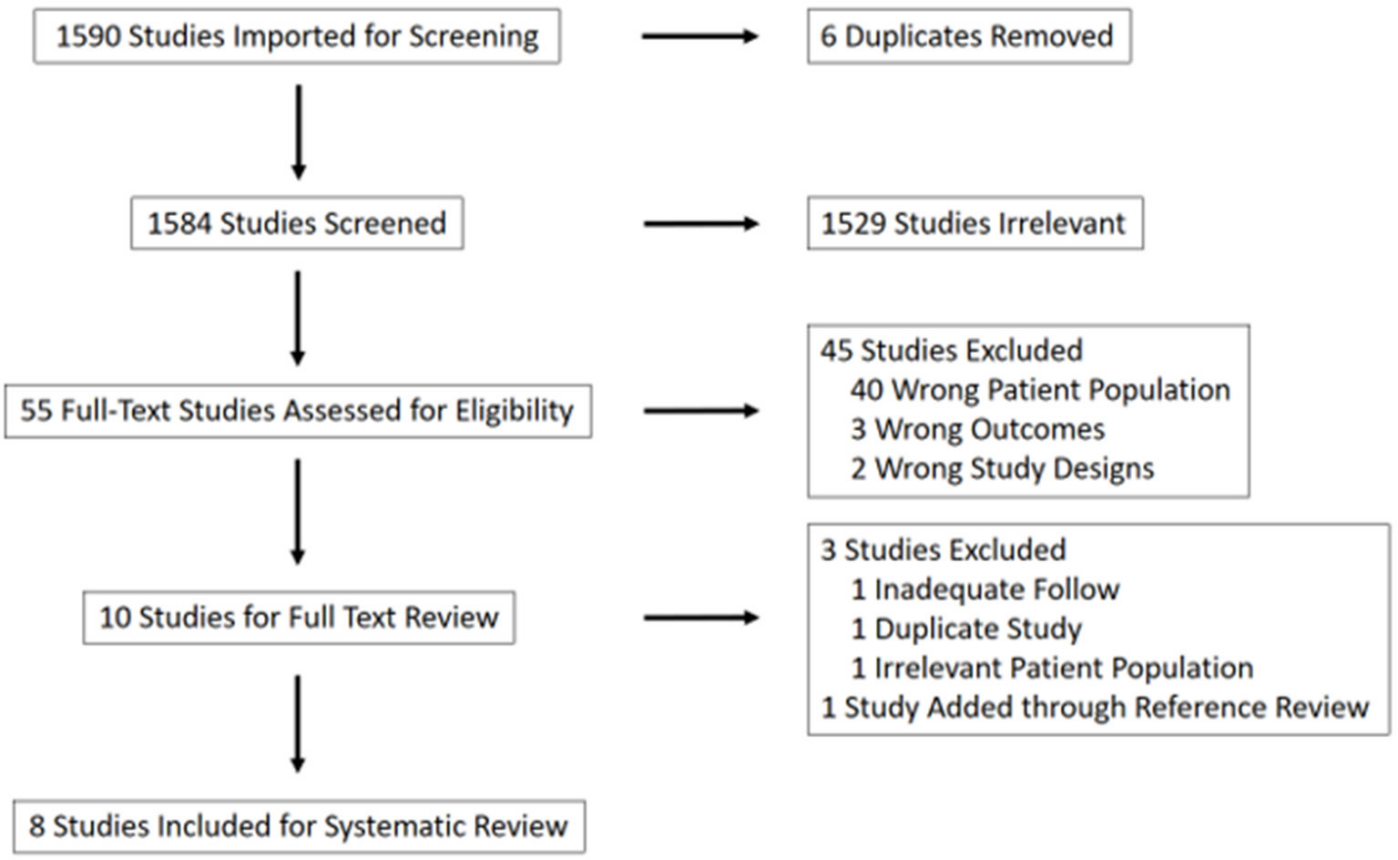
PRISMA flow chart for reference selection.

Using these search terms, 1,584 unique studies were identified. Of these, 1,529 were excluded based on title review. Forty-five of the remaining 55 studies were excluded due to irrelevance after abstract review (40 due to patient population, three due to outcomes, two due to study design). The remaining 10 studies were included for full-text review and pooled analysis (Figure 1).

Of these 10 studies, an additional three were ultimately excluded. The excluded papers included a 20-patient review of pathological characteristics of HCC with BDTT, in which two patients underwent transplantation; one patient was lost to follow-up and the second was the subject of a more recent case report by the same authors which was already included as an independent paper in this study (9, 10). Two papers by Peng et al. provided a review of the same patient, and so the case report with more recent follow-up was included (11, 12). An additional paper was ultimately excluded given that only previously transplanted patients with HCC recurrence in their transplant graft were included, which was considered to be a misrepresentative patient population for this analysis (13).

One additional paper was identified through reference review of the included studies and was added to the analysis (14, 15). This was a multi-center study referenced by Ha et al. (14) and Moon et al. (15) that included seven patients who underwent transplantation for HCC with BDTT. Four of these patients were excluded to avoid duplication given an inability to discern from the text whether they had been included in the aforementioned review by Ha et al. Table 1 summarizes the eight papers that were ultimately included in our analysis.

**Table 1:** Included studies with patient and tumor characteristics (10, 12, 14–19).

**Reference**	**Study type**	**N**	**Age (range)**	**Gender**	**HBV+ history**	**Macro-vascular invasion**	**Micro-vascular invasion**	**Therapy**	**Other HCC therapies**	**Follow-up**	**Notes**
Peng et al. (11, 12)	Retrospective review (1994–2002)	1	53	M	Not specified	Not specified	Not specified	DDLT	None	27 months	15 patients included in review, only one underwent transplant and is included in analysis.
Lee et al. (19)	Retrospective review (1996–2004)	4	57 (45–68)	M	Not specified	Not specified	4/4	LDLT	None	20.6 months (17.6–28.1)	
Xiangji et al. (16)	Retrospective review (1998–2006)	5	Not specified	Not specified	Not specified	Not specified	Not specified	DDLT	None	8 years	2 patients excluded due to aborted transplant.
Liu and Wang (10)	Case report	1	42	M	1/1	Not specified	Not specified	DDLT	Left hepatectomy for poorly differentiated HCC 6 years prior; RFA x 2 for recurrent HCC.	6 years	
Moon et al. (15)	Retrospective review (1994–2011)	5	57.2 (45–70)	Not specified	2/5	1/5	Not specified	4 LDLT 1 DDLT	Two additional patients underwent salvage LDLT for HCC recurrence after prior resection and are included in our analysis.	5.4 years	Four patients excluded due to overlap with Ha et al. study.
Ha et al. (14)	Retrospective review (2000–2009)	14	54.6 (45–62)	10 M 4 F	11/14	3/14	8/14	1 LDLT 13 DDLT	None	10 years	
Kim et al. (17)	Retrospective review (2002–2008)	8	53.1 ± 9.1	M	6/8	Not specified	Not specified	LDLT	None	3 years	
Uylas et al. (18)	Case report	1	51	M	Yes (HBV DNA PCR negative)	0/1	1/1	LDLT	CBD resection w/ Roux-en-Y HJ (suspected cholangiocarcinoma, found to be HCC on frozen).	6 years	

### Patient Characteristics

A total of 39 patients across the eight studies were initially included in our review. Four of these patients were excluded due to duplicity between case series as noted above; an additional two patients who underwent salvage liver transplant after HCC recurrence were, however, included in our analysis (15). Another two patients were excluded because their transplant was aborted (16). This left a total of 35 patients with survival and/or recurrence data that were able to be pooled for analysis. The average age of patients included in the analysis was 51.7 years, and all patients underwent orthotopic liver transplantation from a deceased donor or living donor. Patient characteristics, including previous therapies and HBV status, are reported in Table 1. There was a greater number of male patients reported in the studies, and of the 28 patients for whom hepatitis B data were available, 21 had a positive history.

### Vascular Invasion

There were data about macrovascular invasion reported for 20 patients. Of those, four were noted to have macrovascular invasion. Of 19 patients with data about microvascular invasion available, 13 were noted to have microvascular invasion on pathological examination of their explanted livers. Rates of macro- and microvascular invasion are reported in Table 1.

### Patient Survival and Recurrence

**Figure 2:**
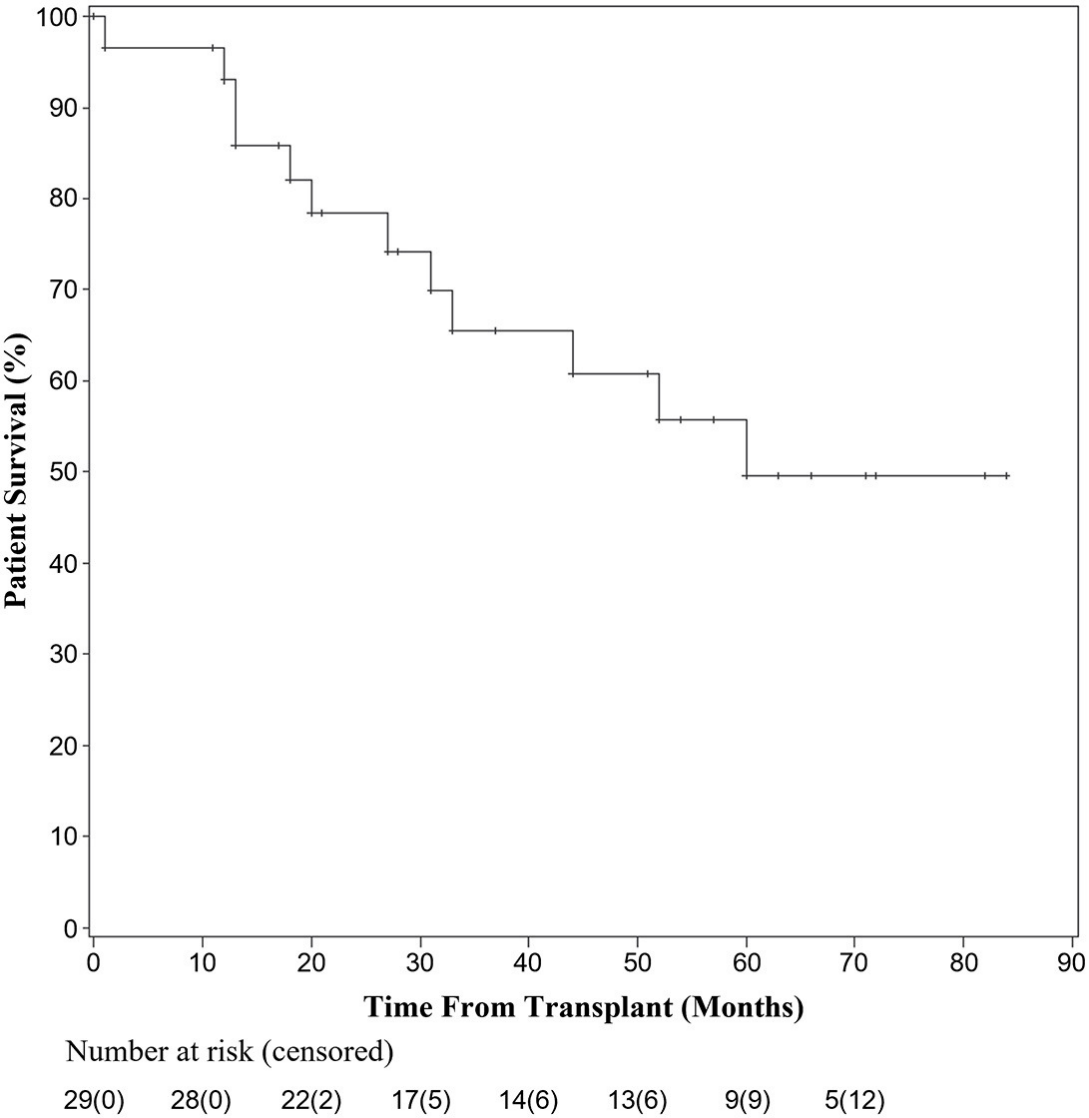
Kaplan-Meier analysis of overall survival after liver transplant for HCC with BDTT.

Overall patient survival is shown in Figure 2. Kaplan-Meier survival analysis was performed after abstracting times to death per patient from each reference. Twenty-nine patients had survival and follow-up data available for analysis. At 1 year, 27 patients undergoing liver transplantation for HCC with bile duct thrombi remained alive. One patient died at 1 month secondary to a gastrointestinal hemorrhage, while the other died 12 months after an initial attempt at resection followed by salvage transplantation. By 36 months 20 were living; with one patient dying from an intraoperative cardiac death while undergoing a pancreaticoduodenectomy for distal bile duct recurrence. The remaining causes of death were not reported, but all deceased individuals had HCC recurrence at the time of death. At 60 months, overall survival was 17 of 29 recipients.

**Figure 3:**
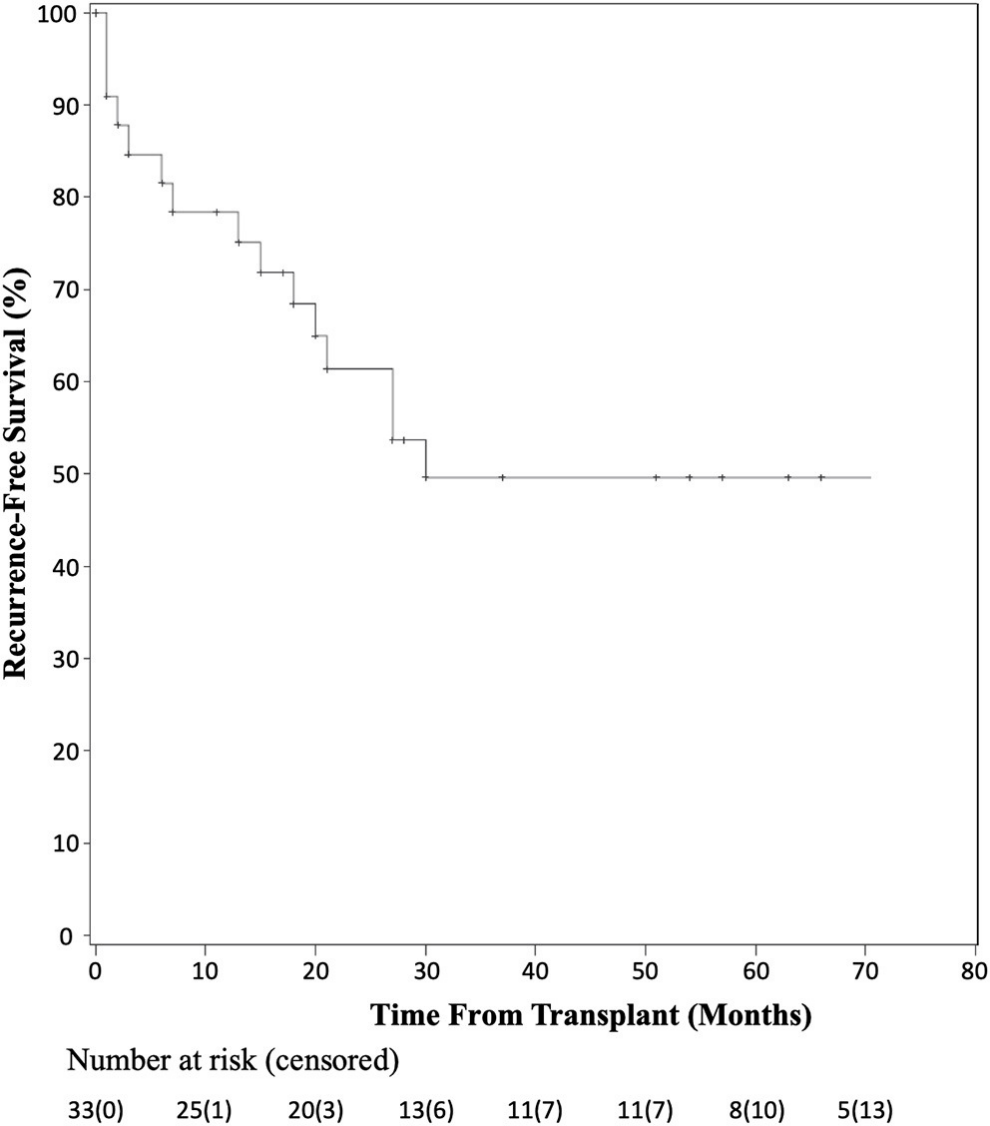
Kaplan-Meier analysis of recurrence-free survival after liver transplant for HCC with BDTT.

Recurrence-free survival data were available for 33 patients as shown by the Kaplan-Meier curve in Figure 3. At 1 year, seven patients had recurrence of their disease and at 3 years, 15 of 33 patients had documented recurrence. Of those patients having recurrence within the first year, five of seven recurred within the first 3 months post-transplant. Detailed patient recurrence and survival data are detailed in Table 2.

**Table 2:** Recurrence and death outcomes per patient (10, 12, 14–19).

**Reference**	**Patient**	**Recurrence**	**Time to recurrence (months)**	**Outcome**	**Time to death or known follow-up (months)**	**Notes**
Peng et al. (11, 12)	1	Yes	27	Dead	27	Whipple for distal bile duct recurrence, death from cardiac arrest intraop
Lee et al. (19)	2	Yes	15	Dead	20	
Lee et al. (19)	3	No	N/A	Alive	17	
Lee et al. (19)	4	No	N/A	Alive	21^*^	
Lee et al. (19)	5	No	N/A	Alive	28	
Xiangji et al. (16)	6	No	N/A	Alive	11^*^	
Xiangji et al. (16)	7	No	N/A	Alive	20^*^	
Xiangji et al. (16)	8	No	N/A	Alive	37	
Liu and Wang (10)	9	No	N/A	Alive	82	
Moon et al. (15)	10	Yes	Unknown	Dead	12	Transplant after prior resection; death noted is from initial resection
Moon et al. (15)	11	Yes	Unknown	Dead	60	Transplant after prior resection; death noted is from initial resection
Moon et al. (15)	12	Yes	20	Dead	44	
Ha et al. (14)	13	No	N/A	Dead	1	Death from GI bleed
Ha et al. (14)	14	Yes	3	Dead	13	
Ha et al. (14)	15	Yes	7	Dead	13	
Ha et al. (14)	16	Yes	18	Dead	18	
Ha et al. (14)	17	Yes	21	Dead	31	
Ha et al. (14)	18	Yes	27	Dead	33	
Ha et al. (14)	19	Yes	30	Dead	52	
Ha et al. (14)	20	No	51	Alive	51	
Ha et al. (14)	21	No	54	Alive	54	
Ha et al. (14)	22	No	57	Alive	57	
Ha et al. (14)	23	No	N/A	Alive	63	
Ha et al. (14)	24	No	N/A	Alive	63	
Ha et al. (14)	25	No	N/A	Alive	71	
Ha et al. (14)	26	No	N/A	Alive	84	
Kim et al. (17)	27	Yes	1	Dead	Unknown	Time to death not specified, excluded from survival analysis
Kim et al. (17)	28	Yes	1	Dead	Unknown	Time to death not specified, excluded from survival analysis
Kim et al. (17)	29	Yes	1	Dead	Unknown	Time to death not specified, excluded from survival analysis
Kim et al. (17)	30	Yes	2	Dead	Unknown	Time to death not specified, excluded from survival analysis
Kim et al. (17)	31	Yes	6	Dead	Unknown	Time to death not specified, excluded from survival analysis
Kim et al. (17)	32	Yes	13	Dead	Unknown	Time to death not specified, excluded from survival analysis
Kim et al. (17)	33	No	N/A	Alive	66	
Kim et al. (17)	34	No	N/A	Alive	72	
Uylas et al. (18)	35	No	N/A	Alive	72	

* *Minimum estimated follow-up*.

## Discussion

Due to practice variability regarding liver transplantation for HCC among centers, and the unclear transplant candidacy of patients with BDTT, this systematic pooled analysis aimed to identify the prognosis of patients with identified BDTT on pathologic examination of their explanted liver. In the current analysis, overall patient survival fell within the first 5 years after transplant, but then stabilized. There were two patient deaths in the first year, four in the second year, three in the third year, one in the fourth year, and two in the fifth year of follow-up. Six additional patients were not included in the survival analysis due to lack of known time to death; however, all six of these patients had recurrence within 13 months of transplant. The 17 remaining patients included in the study had no documented recurrence and the latest reported survival was seven years after transplantation. Similarly, there was a sharp decline in recurrence-free survival in the first 30 months, but stabilization of recurrence-free survival thereafter. Although the causes of death were not reported for each patient among the pooled studies, the recurrence-free data suggest that much of the early mortality seen in patients undergoing liver transplantation was related to recurrence of their disease. Furthermore, of the seven patients that had documented recurrence within the first year after transplantation, five had recurrence within the first 3 months of transplant, highlighting the importance of early surveillance and follow-up for these patients. The current analysis demonstrates that while the rate of recurrence is high in the first 3 years after transplantation for HCC with BDTT, the overall survival at five years post-transplantation is comparable to that seen after transplantation for HCC with macro- or microvascular invasion (20–22).

While liver transplantations regularly performed for advanced HCC according to various criteria, the identification of BDTT on the explanted liver specimen is a relatively rare event. This may be related to the exclusion of many patients from transplant listing based on preoperatively identified radiographic risk factors, thus inferring that those with BDTT may represent a more advanced subgroup of patients. Alternatively, the paucity of data in this population may indicate a lack of awareness within the transplant community as to the implication of BDTT on outcomes after transplantation, which is underscored by the relative rarity of this pathologic finding.

Many centers are expanding criteria for liver transplantation for HCC beyond Milan, for example, the UCSF or Toronto criteria. For instance, Lee et al. recently demonstrated a 63.6% overall survival rate in 11 patients with preoperatively diagnosed portal vein tumor thrombi (PVTT), previously an absolute contraindication to transplant (20). In another study, Aydin and Yilmaz reviewed outcomes in 18 cases of HCC with PVTT at a single center and found a median overall survival of 657.5 days (23). They conclude that liver transplantation should be considered in HCC with PVTT but that initial response to locoregional therapies may serve as an indicator of favorable tumor biology to be considered when selecting patients for transplant.

The present study is limited by several factors. First, there is heterogeneity in the included studies, including varied follow-up time points, differences in pre-transplant HCC therapies, and absence of data regarding viral hepatitis history and pathologic specimen findings. Second, some patients had both HCC BDTT and vascular invasion, which may confound the results. There are data from other groups implicating microvascular invasion as a significant risk factor for recurrence, especially for patients transplanted outside of Milan criteria (21, 22, 24–26). Given the small cohort of patients with BDTT, separating out patients with both biliary and vascular invasion was not possible. Third, patient characteristics and survival were summative, precluding us from correlating patient and tumor characteristics with overall patient survival and recurrence data. This presents a particular challenge in that some of the patients excluded from analysis may have been those with early recurrence or death, which could underpower the study given the low number of patients included in statistical analysis. This would artificially increase the survival presented. Fourth, the biliary invasion was identified on pathological examination of the explanted liver. Therefore, it is unclear if these results are translatable to BDTT identified prior to transplantation. Last, all of the studies included in this systematic review were from centers in Asia, with a high prevalence of HBV among patients undergoing liver transplantation for HCC. It is therefore important to consider generalizability to other patient populations, as the majority of HBV-associated cases of HCC occur in Asia and sub-Saharan Africa (27). Further study is warranted to examine the relationship between HBV status and BDTT in HCC and to assess the generalizability of these findings to other populations.

In conclusion, variability remains in centers' willingness to expand inclusion criteria for liver transplantation in patients with a diagnosis of HCC. This systematic review and pooled analysis identified studies across multiple centers with longitudinal follow-up for patients who underwent liver transplantation for HCC with BDTT present on explanation, and aggregated survival data for this understudied population of patients who are at high risk for recurrence after transplantation. The rates of early recurrence and death are comparable to other HCC subgroups undergoing liver transplantation, namely those with PVTT. Therefore, further studies with pre-operatively identified BDTT are required to determine if this patient population should be candidates for liver transplantation.

## Data Availability Statement

The original contributions presented in the study are included in the article/Supplementary Material, further inquiries can be directed to the corresponding author.

## Author Contributions

SK and AB participated in research design, writing of the paper, and performance of the research. CL participated in writing the paper. MH participated in the performance of the research and contributed new reagents or analytical tools. GL participated in data analysis. DA-A participated in research design and performance of the research. All authors contributed to the article and approved the submitted version.

## Funding

DA-A was supported by the National Institutes of Health [K08-AI155816].

## Conflict of Interest

The authors declare that the research was conducted in the absence of any commercial or financial relationships that could be construed as a potential conflict of interest.

## Publisher's Note

All claims expressed in this article are solely those of the authors and do not necessarily represent those of their affiliated organizations, or those of the publisher, the editors and the reviewers. Any product that may be evaluated in this article, or claim that may be made by its manufacturer, is not guaranteed or endorsed by the publisher.

## Abbreviations

BDTT: bile duct-associated tumor thrombi
CBD: common bile duct
DDLT: deceased donor liver transplant
HBV: hepatitis B virus
HCC: hepatocellular carcinoma
HJ: hepaticojejunostomy
LDLT: living donor liver transplant
PV: portal vein
RFA: radio-frequency ablation.
