# Supplementary material for: Liver Transplantation for Hepatocellular Carcinoma With Bile Duct Tumor-Associated Thrombi: A Systematic Review and Pooled Analysis
Source: Front Transplant. 2022 Apr 29;1:879056. doi: 10.3389/frtra.2022.879056 (PMC11235349; doi:10.3389/frtra.2022.879056)
Supplement: Supplementary file 1 [file Data_Sheet_1.PDF]

SUPPLEMENTARY MATERIAL

Supplementary Figure 1: MeSH Search Term Strategy

Combined: 1685

Duplicates Removed: 1588

Hand-Searched Articles: 2

PubMed: 791

Scopus: 894

Liver[tw] OR hepatocellular[tw]	"Bile Ducts"[Mesh] OR "bile duct" OR ("bile duct" AND thromb*[tw]) OR "portal vein" OR "Portal Vein"[Mesh] OR microvascular[tw] OR biliary[tw] OR "major vascular"[tw]	infiltration[tiab] OR infiltrate{tiab] OR invasion[tiab] OR invasiveness[tiab] OR invade[tiab] OR metastasis[tiab] OR mediatized[tiab] OR metastases[tiab] OR involvement[tia	"Transplantation"[Mesh] OR transplantation[tw] OR transplant*[tw] OR "Organ Transplantation"[Mesh]	"Quality of Life"[Mesh] OR "health related outcome" OR "health related outcomes" OR "quality of life" OR surviv* OR

	OR Bile Duct Neoplasm”[Mesh] OR “hepatic duct”	b] OR involving[tiab] OR thromb*[tiab]		accept*[tw]

PubMed 10/15/2020

(((("Quality of Life"[Mesh] OR "health related outcome" OR "health related outcomes" OR "quality of life" OR surviv* OR accept*[tw]) AND ("Transplantation"[Mesh] OR transplantation[tw] OR transplant*[tw] OR "Organ Transplantation"[Mesh])) AND (infiltration[tw] OR infiltrate{tw] OR invasion[tw] OR invade[tw] OR metastasis[tw] OR involvement[tw] OR involving[tw] OR metiastized[tw] OR metastases[tw])) AND ("Bile Ducts"[Mesh] OR "bile duct" OR "bile ducts") OR "portal vein" OR "Portal Vein"[Mesh])) AND ((("Carcinoma, Hepatocellular"[Mesh]) OR "Carcinoma, Hepatocellular/transplantation"[Mesh]) OR "Liver Neoplasms"[Mesh] OR (liver[tw] AND (cancer[tw] OR neoplasm[tw] OR tumor[tw] OR tumour[tw] OR malignancy[tw] OR malignancies[tw] OR malignant[tw] OR carcinoma[tw]))) 5,273 results

(((("Quality of Life"[Mesh] OR "health related outcome" OR "health related outcomes" OR "quality of life" OR surviv* OR accept*[tw]) AND ("Transplantation"[Mesh] OR transplantation[tw] OR transplant*[tw] OR "Organ Transplantation"[Mesh])) AND (infiltration[tw] OR infiltrate{tw] OR invasion[tw] OR invade[tw] OR metastasis[tw] OR involvement[tw] OR involving[tw] OR metiastized[tw] OR metastases[tw])) AND ("Bile Ducts"[Mesh] OR "bile duct" OR "bile ducts") OR "portal vein" OR "Portal Vein"[Mesh]))

AND ((("Carcinoma, Hepatocellular"[Mesh]) OR "Carcinoma, Hepatocellular/transplantation"[Mesh]) OR "Liver Neoplasms"[Mesh] OR (liver[tw] AND (cancer[tw] OR neoplasm[tw] OR tumor[tw] OR tumour[tw] OR malignancy[tw] OR malignancies[tw] OR malignant[tw] OR carcinoma[tw]))) AND adult ---3700+

REMOVED QOL ETC

(((("Transplantation"[Mesh] OR transplantation[tw] OR transplant*[tw] OR "Organ Transplantation"[Mesh])) AND (infiltration[tw] OR infiltrate{tw] OR invasion[tw] OR invade[tw] OR metastasis[tw] OR involvement[tw] OR involving[tw] OR metastasized[tw] OR metastases[tw])) AND ("Bile Ducts"[Mesh] OR "bile duct" OR "bile ducts" OR "portal vein" OR "Portal Vein"[Mesh])) AND ((("Carcinoma, Hepatocellular"[Mesh]) OR "Carcinoma, Hepatocellular/transplantation"[Mesh]) OR "Liver Neoplasms"[Mesh] OR (liver[tw] AND (cancer[tw] OR neoplasm[tw] OR tumor[tw] OR tumour[tw] OR malignancy[tw] OR malignancies[tw] OR malignant[tw] OR carcinoma[tw]))) AND adult. 2000-2020---176 results

Remove adult at end—2000-2020 in English—398 results

Updated October 16, 2020

((((("Carcinoma, Hepatocellular"[Mesh]) OR "Carcinoma, Hepatocellular/transplantation"[Mesh]) OR "Liver Neoplasms"[Mesh] OR liver[tw] OR (liver[tw] AND (cancer[tw] OR neoplasm[tw] OR tumor[tw] OR tumour[tw] OR malignancy[tw] OR malignancies[tw] OR malignant[tw] OR carcinoma[tw])) AND

(english[Filter])) AND ("Bile Ducts"[Mesh] OR "bile duct" OR "bile ducts") OR "portal vein" OR "Portal Vein"[Mesh] OR microvascular[tw] OR biliary[tw] AND (english[Filter]))) AND (infiltration[tw] OR infiltrate{tw] OR invasion[tw] OR invade[tw] OR metastasis[tw] OR mediatized[tw] OR metastases[tw] OR involvement[tw] OR involving[tw] OR thrombus[tw] OR thrombi[tw] AND (english[Filter]))) AND ("Transplantation"[Mesh] OR transplantation[tw] OR transplant*[tw] OR "Organ Transplantation"[Mesh] AND (english[Filter])) AND adult. 2000-2020, English--943 Results

October 16, 2020 2nd try

(((("Carcinoma, Hepatocellular"[Mesh] OR "Carcinoma, Hepatocellular/transplantation"[Mesh] OR "Liver Neoplasms"[Mesh] OR liver[tw]) OR (liver[tw] AND (cancer[tw] OR neoplasm[tw] OR tumor[tw] OR tumour[tw] OR malignancy[tw] OR malignancies[tw]OR malignant[tw] OR carcinoma[tw]))) AND ("Bile Ducts"[Mesh] OR "bile duct" OR "bile ducts" OR "portal vein" OR "Portal Vein"[Mesh] OR macrovascular[tw] OR biliary[tw])) AND (infiltration[tw] OR infiltrate[tw] OR invasion[tw] OR invade[tw] OR metastasis[tw] OR metastasized[tw] OR metastases[tw] OR involvement[tw] OR involving[tw] OR thrombus[tw] OR thrombi[tw])) AND ("Transplantation"[Mesh] OR transplantation[tw] OR transplant*[tw] OR "Organ Transplantation"[Mesh]) AND (prognosis[tw] OR prognostic[tw] OR survival[tw] OR "long term" OR "long-term" OR outcome[tw] OR outcomes[tw]). 2000-2020/English 683 results

Or these two on Oct 23, 2020 = 791 items

Take out transplantation concept—4090 results. (Waiting to hear from DA on what to do).

Scopus Oct 23, 2020

894 document results

(((TITLE-ABS-KEY ({Hepatocellular Carcinoma} OR {liver cancer} OR (liver AND (cancer OR tumor OR tumour OR carcinoma OR neoplasm OR malignan*))))) AND (transplant*)) AND (TITLE-ABS-KEY ({bile duct} OR {bile ducts} OR {portal vein} OR macrovascular OR biliary)) AND (TITLE-ABS-KEY (prognosis OR prognostic OR survival OR {long term} OR {long-term} OR outcome OR outcomes))) AND (infiltration OR infiltrate OR invasion OR invade OR metastasis OR metastasized OR metastases OR involvement OR involving OR thrombus OR thrombi) AND NOT resect* AND (LIMIT-TO (PUBSTAGE , "final")) AND (LIMIT-TO (DOCTYPE , "ar") OR LIMIT-TO (DOCTYPE , "re")) AND (LIMIT-TO (PUBYEAR , 2020) OR LIMIT-TO (PUBYEAR , 2019) OR LIMIT-TO (PUBYEAR , 2018) OR LIMIT-TO (PUBYEAR , 2017) OR LIMIT-TO (PUBYEAR , 2016) OR LIMIT-TO (PUBYEAR , 2015) OR LIMIT-TO (PUBYEAR , 2014) OR LIMIT-TO (PUBYEAR , 2013) OR LIMIT-TO (PUBYEAR , 2012) OR LIMIT-TO (PUBYEAR , 2011) OR LIMIT-TO (PUBYEAR , 2010) OR LIMIT-TO (PUBYEAR , 2009) OR LIMIT-TO (PUBYEAR , 2008) OR LIMIT-TO (PUBYEAR , 2007) OR LIMIT-TO (PUBYEAR , 2006) OR LIMIT-TO (PUBYEAR , 2005) OR LIMIT-TO (PUBYEAR , 2004) OR LIMIT-TO (PUBYEAR , 2003) OR LIMIT-TO (PUBYEAR , 2002) OR

LIMIT-TO (PUBYEAR , 2001) OR LIMIT-TO (PUBYEAR , 2000)) AND (LIMIT-TO (LANGUAGE , "English"))
